# Process of adhesion of the Adequate Childbirth Program to improve obstetric care in private hospitals in Brazil

**DOI:** 10.1186/s12978-022-01542-3

**Published:** 2023-01-06

**Authors:** Débora Cecília Chaves de Oliveira, Andreza Rodrigues, Lucia Regina de Azevedo Nicida, Maysa Luduvice Gomes, Jacqueline Alves Torres, Elyne Montenegro Engstrom

**Affiliations:** 1 Programa de Pós-Graduação em Saúde Pública, Escola Nacional de Saúde Pública, Fundação Oswaldo Cruz, Rua Bento Lisboa, nº 80, Apt. 303, Bairro Catete, Rio de Janeiro, RJ 22221-010 Brazil; 2 Saúde Coletiva Pelo Instituto Nacional de Saúde da Mulher, da Criança E Do Adolescente Fernandes Figueira, Fundação Oswaldo Cruz, Rio de Janeiro, RJ Brazil; 3 Saúde Coletiva Pelo Instituto Nacional de Saúde da Mulher, da Criança e do Adolescente Fernandes Figueira, Fundação Oswaldo Cruz, Rio de Janeiro, RJ Brazil; 4 Escola de Enfermagem Anna Nery UFRJ, Rio de Janeiro, RJ Brazil; 5 Technical Director do Institute for Healthcare Improvement, Rio de Janeiro, RJ Brazil; 6 Saúde Coletiva Pela Escola Nacional de Saúde Pública, Fundação Oswaldo Cruz, Rio de Janeiro, RJ Brazil; 7 Departamento de Ciências Sociais, Escola Nacional de Saúde Pública, Rio de Janeiro, RJ Brazil; 8 Escola de Enfermagem Anna Nery, Universidade Federal do Rio de Janeiro, Rio de Janeiro, RJ Brazil; 9 Faculdade de Enfermagem da UERJ, Rio de Janeiro, RJ Brazil

**Keywords:** Diffusion inovation, Innovation, Healthcare, Quality improvement

## Abstract

**Background:**

In 2015, a quality improvement project called “Projeto Parto Adequado-PPA” was implemented in Brazilian private hospitals to reduce unnecessary high rates of cesarean sections. This study aimed to analyze the decision-making process of managers and care leaders to adhere to the PPA.

**Methods:**

The Healthy Birth study is evaluative research that used mixed methods to evaluate the implementation and effects of the Adequate Childbirth Program in 12 hospitals that participated in the program. Eight out of 12 hospitals were selected for a qualitative approach. We interviewed ten managers and 24 care leaders from July to October 2017. The interviews were transcripted, and data was systematized using the MaxQda software, with Thematic Content Analysis, to identify the facilitators and barriers for adherence to the Adequate Childbirth Program. We used the conceptions of the Diffusion of Innovation as an analytical reference.

**Results:**

The main reasons to adhere to the Adequate Childbirth Program were the absence of other quality improvements programs in Brazilian private services using multifaceted interventions, social and market status for participating; commitment to quality of care; and the possibility of structural reforms related to the Adequate Childbirth Program implementation. In addition, inviting hospital influencers to learn about the objectives and intentions of the project before joining was considered an important strategy to motivate hospitals.

**Conclusion:**

Social, cultural, and economic constructs motivated adherence. The invitation strategy used by the Adequate Childbirth Program coordination, through socially respected members in Brazil, such as doctors, was highly valued by the leaders of the hospital team and encouraged adherence to the Program.

## Plain language summary

In 2015, a quality improvement program, called the "Adequate Childbirth Program" (PPA), was recommended by the National Supplementary Health Agency so that Brazilian private hospitals could, through a change in the care model, reduce unnecessary cesarean sections and stimulate the natural and safe process in childbirth. The process of adhering to the PPA was studied in this article. Ten managers and 24 assistant leaders were interviewed in 08 private hospitals in Brazil. The main reasons for joining the PPA were the absence of other quality improvement programs; for being a program considered necessary in the country's midwifery, which could increase the prestige of hospitals in the view of women and families; for proposing an improvement in the quality of care; and for stimulating of structural and ambiance reforms. Social, cultural, and economic values motivated hospital adherence. The invitation strategy used by the PPA coordination, through socially respected members in Brazil, such as medical professionals, was highly valued by the hospital's leaders and encouraged adherence to the Program.

## Background

In Brazil, the logic of health system organization, typical of a capitalist state, conjugates different practices and care models. The Brazilian Unified Health System (SUS) combines public and private subsectors [1]. The characteristics of the private subsector in this context highlight a model of care based on the medical figure, with a diversity of interests involved in resource financing and application and the effect of the financial market and work for an organization on the care process [2].

Unsatisfactory health outcomes, such as high cesarean rates in the Brazilian private sector, require sophisticated solutions with great inventive potential. In the private sector, cesarean section accounts for 83 percent of births [3], a statistic that does not match the World Health Organization (WHO) safety indication of 10–15 percent [4, 5], other studies [6, 7], or even the adjusted cesarean section rate for Brazil of 25–30 percent [8].

In 2006, an extensive movement of women, called “Parto do Princípio”, began the struggle for rights related to childbirth and birth in Brazil. Since then, it has been present in the fight against obstetric violence and is presented essentially in the scenario of dialogue in the health system to adopt measures that quality care and reduce the high rates of cesarean sections [9].

The “Parto do Princípio” movement presented a dossier to the Federal Public Ministry proposing changes in the Brazilian obstetric private sector in 2006 [9]. In 2010, the Federal Public Ministry filed a Public Civil Action against the National Supplementary Health Agency (ANS), which demanded the regulation of private obstetric services, complying with the propositions inserted in the dossier carried out by the women's movement [10].

In this sense, such reality induced in 2015 the creation of the Adequate Childbirth Program (Projeto Parto Adequado—PPA), which is an intervention proposed by the National Agency for Supplementary Health (ANS), to improve delivery and birth care in Brazilian private hospitals. In which it proposed to identify innovative and viable models of care, based on successful experiences and scientific evidence, in an attempt to reduce the potential increase of unnecessary risks of cesarean sections, insecurity, and experiences that do not reveal the empowerment and protagonism of women in the scenario of labor and birth [11].

Considered an intervention (the PPA) with high innovative power to promote a new model of care in institutions (the innovation), the main focus of the program was to reduce the high rates of cesarean sections (CS), promote normal birth and qualified care from prenatal to postpartum, through interventions in the care process [3]. Thus, the promotion of this new model of care corresponds to the transformation of a static or dynamic scenario through the use of technologies, processes, and interventions; political, social, and institutional [12–15] incorporations, which could be carried out incrementally to what already existed in the context; but could also be implemented radically [15], requiring the extinction of the previously existing obstetric care model.

The appropriation of a new care model during the adherence decision-making process probably went through people who took into account possible impacts, such as the degree of uncertainty when adhering; the power to solve problems; economic, sustainable, and cultural factors involved in the process, and goals to be achieved after adherence [13]. Other factors might justify the expenditure of time, knowledge, and money in implementing an intervention [15].

**Fig. 1:**
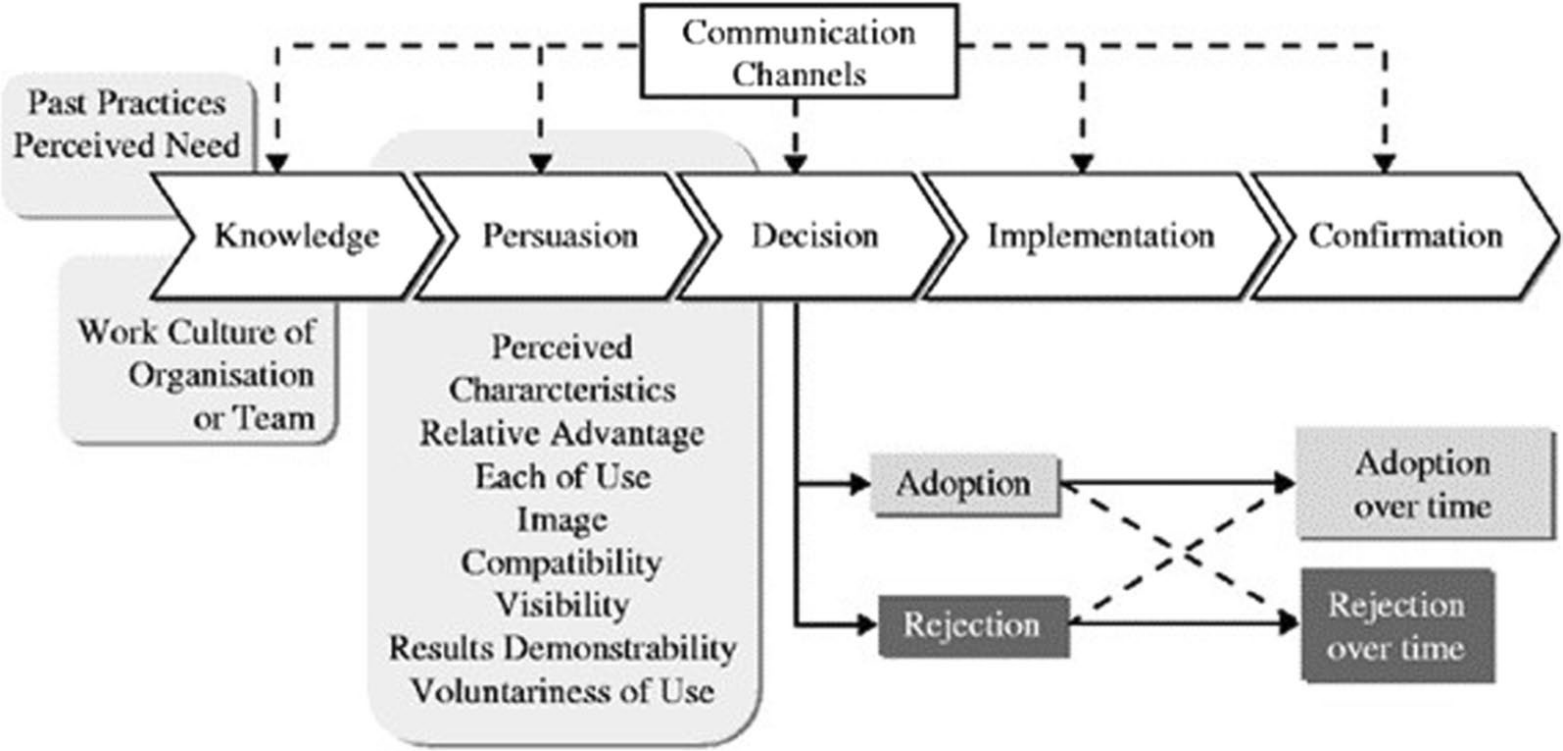
A model of Five Stages in the Innovation-Decision Process. Source: Diffusion of Innovations, Fifth Edition by Everett M. Rogers. Copyright (c) 2003

Usually, innovations pass through a communication transmission channel, in which opinion leaders are potent instruments in the diffusion to their peers, offering greater assurance in the practical use of innovation by the collective during its implementation [15]. Thus, adopting an innovation happens when information is transmitted and processed to reduce the uncertainty of adopting it or not. For Rogers [15], this process usually happens linearly over some time and involves 05 (five) stages: 1. Knowledge, 2. Persuasion, 3. Decision, 4. Implementation, and 5. Confirmation (Fig. 1).

According to Rogers [15], to make a decision, preconditions prior to the innovation provoke the initial desire to join. In addition, he mentions that knowing the innovation well allows understanding how it works and seeks to reduce its uncertainties. In the persuasion phase, the individual or the organization builds a favorable or unfavorable attitude toward adopting the innovation. On the other hand, the decision phase involves the act of accepting or rejecting the innovation. Its adoption refers to the choice to use it, and rejection means that adopting it is not the best choice. If the group, person, or organization accepts the innovation, it continues to the next step, which is the execution of the innovation. Implementation is when the person or organization puts the innovation into practice. At this stage, there may be what Rogers calls reinvention, which is the adaptation of the innovation to the local context. There is a reevaluation in the last stage of confirmation to continue or not with the innovation.

Understanding how hospitals in the private subsystem learned, were motivated and decided to enroll in the Adequate Childbirth Program to innovate their obstetric care model will allow us to understand the limits and achievements in the ongoing process and after implementation. Thus, this article aims to analyze the decision-making process of care managers and leaders to enroll in the PPA. This paper focuses on the pre-innovation conditions and the first three stages of the innovation adoption process described by Rogers (Fig. 1).

## Methods

Recognizing the importance of the Adequate Childbirth Program intervention to the health of Brazilian women and children, the National School of Public Health (ENSP)—Oswaldo Cruz Foundation (FIOCRUZ) proposed an evaluation titled “Healthy Birth: a prospective study to evaluate the implementation and effects of a multifaceted in-hospital intervention.” In order to analyze the implementation and effects of the PPA in a sample of 12 hospitals utilizing mixed methods analysis [3].

The survey was carried out at two different times for data collection. The first [M1] focused on assessing the degree of implementation and the intervention’s effect; the second moment [M2] focused on assessing the sustainability of the intervention. More details on data collection, contextual aspects, and protocols established by the “Healthy Birth” survey can be found in Torres et al. [3] and Domingues et al. [16].

### Study type and subsample

**Fig. 2:**
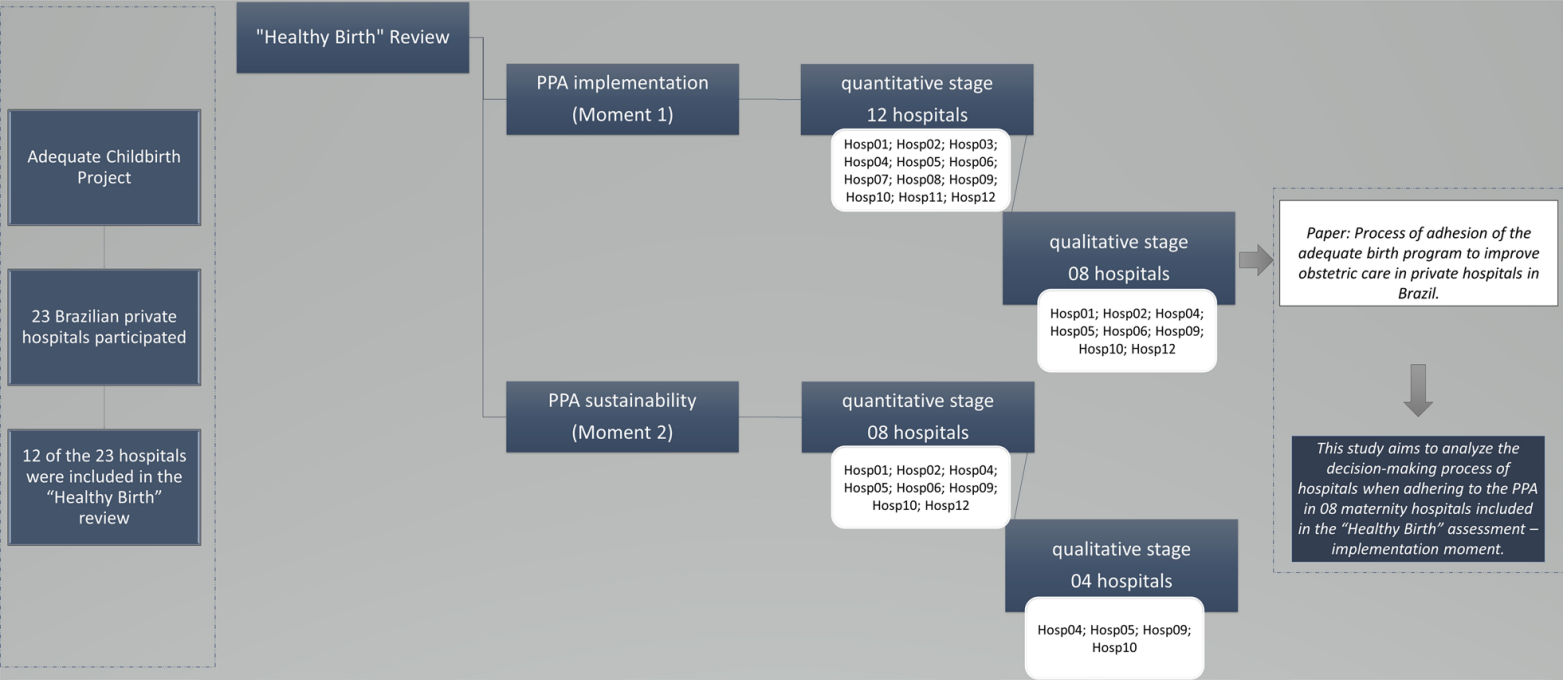
Selection process of participating hospitals. Created by the authors

Integrated to this evaluation, this article develops a single case study with an exploratory qualitative approach, which chooses the intervention Projeto Parto Adequado as a case under analysis. For this qualitative analysis, a subsample of 08 (eight) hospitals (Hosp01; Hosp02; Hosp04; Hosp05; Hosp06; Hosp09; Hosp10; Hosp12) was intentionally selected (Fig. 2). The inclusion criteria for this subsample were the location of the maternity hospital according to the country’s macro-region, the type of maternity unit belonging or not to health insurers, and the maternity’s performance in achieving goals to reduce cesarean sections (data provided by ANS/PPA monitoring). We excluded four hospitals (Hosp03; Hosp07; Hosp08; Hosp11) due to geographic location and management [3].

### Selection of participating professionals

The technique used to select such participating professionals was Snowball sampling [17], a non-probabilistic sampling that focuses on reference chains, used mainly in exploratory studies. Identifying key informants or seeds for the interviews began with the managers, who identified the project leaders/coordinators in the hospital. We selected 34 professionals directly linked to the management and coordination of hospitals, 10 (ten) managers, and 24 leaders/coordinators. There was no refusal from the participants in any of the hospitals. Managers were considered actors with greater decision-making power, and leaders/coordinators as those who directly support managers in the work decision-making process.

### Data collection scenario

The training of the interviewers who carried out the data collection was carried out in two ways: in person for 02 (two) coaches and remotely for another 02 (two) coaches. In these trainings, there was a reading of the instrument, presentation of techniques for conducting interviews, field observations, and the importance of records.

Regarding the characterization of the 04 (four) interviewers who carried out the data collection: they were all women with academic training in Nursing, Midwifery, and History. The historians were researchers in public health; the nurse and the Midwife were researchers in women’s health; all had previous experience with data collection for research. These interviewees were drawn to the field mainly because they were already involved in women’s health research or care practice. They were presented as such to the interviewees.

Before entering the field for data collection, pilot tests of the scripts were carried out in a maternity hospital in Rio de Janeiro/Brazil, part of the Adequate Childbirth Project. However, this maternity hospital was excluded from the sample of hospitals in the Nascer Saudável evaluation. This phase made it possible to make adjustments and validate the interview scripts. Furthermore, the research team established a previous contact connection with the management of the institutions to agree on the dates on which the interviewers would be placed in the field for data collecting and any ethical issues that would be released.

The immersion in the hospitals took place during a period of 05 (five) days, from July to October 2017, in which the research team carried out interviews with managers coordinators/leaders. A semi-structured script was used for managers and leaders/coordinators, which included the following axes: decision process; deployment strategies; participation of the assistance team; women’s participation; monitoring; strategy results. This script was developed for Healthy Birth research [3].

The interviews took place in the hospitals themselves; privacy was maintained in rooms that could have closed doors and away from other people, with only the interviewer and the interviewee remaining in the room. The interview length (average of forty minutes) was not pre-determined and varied according to each interviewee's level of involvement in the project and subjective features. The interviews were audio-recorded. During this process, there was no need to repeat interviews. Field notes were taken at the end of each hospital field.

Subsequently, the interviews were transcribed by an independent professional. Such transcripts were not returned to the participants for evaluation, comments, and corrections. However, for the order of internal validation of the transcripts, there was a review by the research team.

About data saturation, it is worth noting that qualitative research, unlike quantitative research, is not based on how many individuals should be heard but on the intensity of the phenomenon and scope to which the actors are linked to the researched object. This emphasizes the need to fine-tune the interviewee selection process. However, the recommended minimum of at least 20–30 interviews was used for qualitative investigations [18].

### Data storage

Work was carried out on constructing and analyzing the database in the MaxQda software [19]. During the database construction stage, the interviews were inserted, organized, and encrypted in the software according to the moment of the evaluative research, the ID of the hospital where the interviewee was linked, the professional category belonging, and finally, a professional identifier number. All citations in this article only include the professional position held by the interviewed leadership, in order to avoid possible identification.

### Data analysis

According to Uwe Flick [20], the data were submitted to Thematic Content Analysis. Focusing on the axis of the semi-structured decision-making process, the following dimensions were explored: whether the maternity hospital was already developing some initiative related to childbirth care before the PPA; how maternity got to know the PPA; what motivated the participation of maternity in the PPA; and finally, who decided for maternity to participate in the PPA.

The open categorization was then carried out, generating broad segments. With the refinement of these segments, a list of codes was generated, a step called axial coding. An inductive association was made to create the categories [20]. The entire data analysis course was permeated by the theoretical framework of the Diffusion of Innovation by Everett M. Rogers [15].

Although there was no feedback from the participants regarding the findings, for the interpretation of the data, the interviewees’ speeches and their respective coding were validated by members of the research group, in which there were also reflexive co-participations on the interpretive procedures of the entire analytical phase [20].

As a methodological guide for qualitative research, it used the consolidated criteria for reporting qualitative research (COREQ) [21].

## Results

### Characterization of the participants

The 34 professionals interviewed were directly related to the management and coordination of hospitals, 10 (ten) managers, and 24 leaders/coordinators. These professionals inserted in 08 (eight) hospitals, which distributed in 03 (three) regions of the country (Northeast, Southeast, and South) and 06 (six) Brazilian states (Ceará, Rio de Janeiro, São Paulo, Espírito Santo, Rio Grande do Sul and Santa Catarina). Of the 10 managers, 80% (n = 08) are male and 60% (n = 06) are doctors. Regarding the leaders/coordinators, the majority are female 54.2% (n = 13) and 58.3% (n = 14) are physicians (Table 1).

**Table 1:** Characterization of the participants (managers and leaders/coordinators) according to hospitals

	Hosp01	Hosp02	Hosp03	Hosp04	Hosp05	Hosp06	Hosp07	Hosp08	All hospitals
Country region	Northeast	Southeast	Southeast	Southeast	South	South	Southeast	Southeast	03 Brazilian regions
Country state	Ceara	Rio de Janeiro	São Paulo	Espírito Santo	Santa Catarina	Rio Grande do Sul	São Paulo	São Paulo	06 Brazilian states
Managers	02	01	01	01	01	01	01	02	10 (29.4%)
Leaders/Coordinators	03	03	02	03	04	03	03	03	24 (70.6%)
*Position “Manager” by gender n* = *10*									
Managers—female	0	01	0	0	0	0	0	01	02 (20%)
Managers—male	02	0	01	01	01	01	01	01	08 (80%)
*Position “Leader/Coordinator” by gender n* = *24*									
Leaders/Coordinator -female	02	02	01	03	01	02	01	01	13 (54.2%)
Leaders/Coordinator -male	01	01	01	0	03	01	02	02	11 (45.8%)
*Position “Manager” by professional category n* = *10*									
Medical managers	01	0	01	01	01	01	01	0	06 (60%)
Non-medical managers	01	01	0	0	0	0	0	02	04 (40%)
*Position “Leader/Coordinator” by professional category n* = *24*									
Medical leaders/coordinator	02	02	01	02	03	0	02	02	14 (58.3%)
Non-medical leaders/coord	01	01	01	01	01	03	01	01	10 (41.7%)
Total interviewees: 34									

Source: Created by the authors

**Fig. 3:**
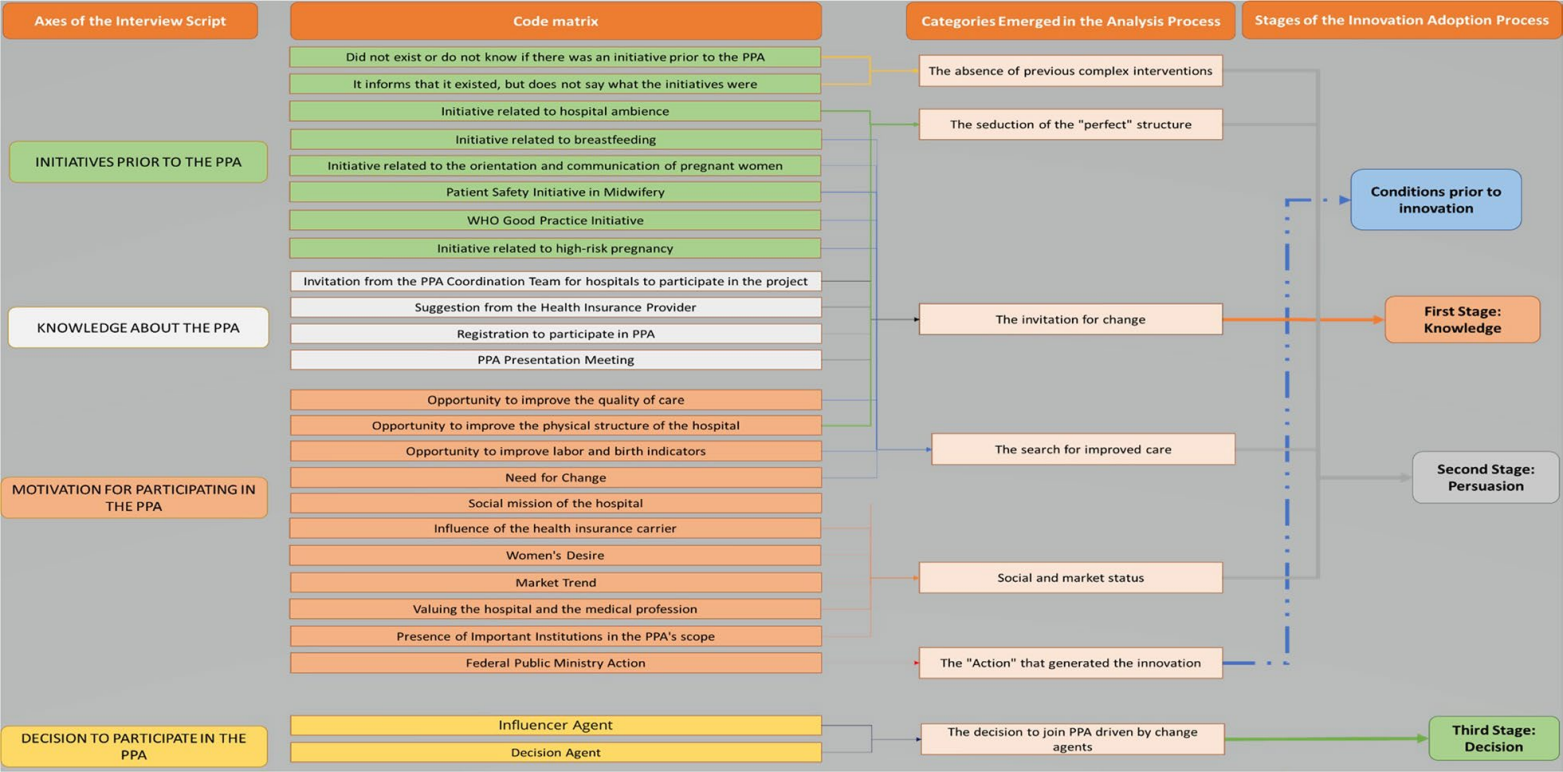
Data categorization process and the correlation with pre-innovation conditions and the first three stages of the PPA adoption process. Created by the authors

The categories that emerged in the analysis process intertwined with the Diffusion of Innovations Theory (Fig. 3).

- Conditions Prior to Innovation: The “action” that generated the innovation.
- First Stage—Knowledge: The invitation to change.
- Second Stage—Persuasion: The absence of previous interventions; The seduction of the “perfect” structure; The search for improved care; Social and market status.
- Third Stage—Adhesion: The decision to join PPA is driven by change agents.

## Conditions prior to the innovation

### The “Action” that generated the innovation

When Brazil, through the hegemonic model of care, produces the cesarean delivery route as an almost predominant form of birth, which does not favor maternal and neonatal morbidity and mortality rates, it is necessary to think of strategies to change this experience.

*“It was really that high cesarean rate. [The] Public Ministry also, made a participation, visited, questioned our numbers. It was not interesting for the hospital to have that high number.” (leader/Coord).*

One of the responses to the action of the Public Ministry was the proposition, by ANS, of a program to improve the quality of obstetric and neonatal care in the supplementary service through institutional, scientific, and methodological support to hospitals that wished to reorganize concerning prenatal care, delivery and puerperium. The Adequate Childbirth Program fostered the reduction of CS without clinical indications and possible adverse events resulting from inadequate childbirth through the proposition of a new model of care.

*“[…] the Program was presented due to a demand that arose at the time, generated by the Federal Public Ministry. They started a strong campaign and fined the hospitals regarding obstetric violence, and we had the presence, here, of the Federal Public Ministry, and an agreement that the hospital made with the Federal Public Ministry was to be part of the Program […]” (Leader/Coord).*

This situation was the impetus for a feasible and essential shift in obstetric care in the private network, and the ANS’s backing encouraged hospitals to follow the program.

## First stage: knowledge

In the first stage of innovation, called knowledge, Rogers describes it as “when an individual is exposed to the existence of the innovation and gains some understanding of how it works” [15]. In analyzing the interviews, the category produced:

### The invitation for change

The ANS purposefully invited private hospitals with the most significant delivery volume in Brazil1 to an introductory conference where the scope and objectives of the PPA would outline [3]. This method had a good influence on the appreciation of these services and their representatives.

*“We received an invitation, directly from the Program Organizing Committee, which is IHI, ANS, and Einstein, inviting the superintendent, our CEO (Chief Executive Office), for a meeting […] where the program, objectives […] would present the program and its objectives” (Manager).*

After the meeting to present the PPA, hospitals interested in joining the program registered an adhesion form with ANS, justifying their desire to participate. Soon after, the PPA coordination team would select the hospitals for the program.

*“[…] because you had to apply to view the hospitals that were going to be picked, and they [the two physicians who attended the conference] did the application, and the hospital was chosen.” (leader/Coord)*

The desire to change the model of care may or may not have preceded the exposure to the operation of the PPA. However, knowledge of the program through the meeting may have been paramount to raise fundamental questions, such as: how the PPA would work in the maternity ward; how the program would work properly; what would be the costs for hospitals; what would be the support network involved in the change of model, among other issues. According to the “knowledge” stage of the Theory of Diffusion of Innovations [15], this allows to reduce uncertainties in the adoption and sustain innovation for an expected result, reducing its rejection or discontinuation.

## Second stage: persuasion

The second stage, called persuasion, is described by Rogers [15] as when the individual exposed to the innovation builds a favorable or unfavorable attitude towards it. Considering that, in this study, we interviewed only individuals who “apparently” built a favorable attitude to the PPA, because they were institutions that had already accepted the adhesion term at the time of the research, we will demonstrate what, probably, these individuals used strategies to persuade their peers to adopt the PPA. This stage of the theoretical referential was composed of four categories of analysis: the absence of previous complex interventions; social and market status; the seduction of the “perfect” structure; and the search for improved care.

### The absence of previous complex interventions

The ANS employed the lack of past interventions or the presence of non-systematized interventions with the potential to change the model of care as a persuasive object for program adherence.

*“We used to do our daily routine. Obstetricians who did births introduced news, which we implanted, but it was not a systematic process. That was not the situation.” (Leader/Coord)*

*“It was all the idea of Proper Childbirth. There were isolated ideas (to implement change projects in the hospital), so I thought about some things. I had an idea in the pediatrics, breastfeeding part, other isolated ideas […], but we (the hospital leaders) did not talk about it (before the PPA).” (leader/Coord).*

Some PPA recommendations, including the participation of management for change, the enhancement of professional, functional performance, the inclusion of women and families in care, and the monitoring of indicators, may have helped the PPA's acceptance. These incitements that, presented during the meeting of presentation of the PPA, may have provided the actors that represented the hospitals and that still did not have structured interventions to change the care model, to adhere to the PPA.

### Social and market status

The need to belong to representative classes permeates our sociocultural construction. Nevertheless, the social and market status exposed in the speeches reminds us of the need hospitals have in occupying places considered necessary by the social construct, whether by women, families, and social movements or by professionals, scientific academia, and imposing organizations in obstetrics.

*“[…] understanding that women currently seek this issue of natural childbirth, of proper childbirth. Furthermore, the hospital has always sought to be within this profile. So I believe that when it went into this field, it has this goal of corresponding to what women today expect from birth.” (leader/Coord).*

*“[…] but as we are in the maternity market, we have every interest in doing the best possible, with all the quality, so, we thought it was important to participate […]” (Manager)*

In this sense, the professionals realized that being aligned to PPA could also open paths for partnerships with other agents involved in the program's composition. Besides the representation of the National Supplementary Health Agency, the professionals pointed out the presence of the Hospital Albert Einstein and the Institute Healthcare Improvement (IHI) as necessary for the adhesion to PPA.

*“When you see the ANS, Einstein, and the IHI, references to quality processes in health care, wow! This group is not kidding. Let us learn! So we had every opportunity, we had a partnership with Einstein, we had open doors at Einstein, our doctors and our nurses participated in the training, realistic simulations, all that there, this is unique.” (leader/Coord).*

*“(…) the Program seemed quite interesting, both from the medical point of view and also, that it adds value to the institution (…)”. (Manager)*

### The seduction of the “perfect” structure

Undeniably, to ensure a good quality of care during labor and birth, the physical structure of the maternity ward must be taken into account. Quality of care includes clinical effectiveness, patient safety, and efficiency of care, factors based on indicators such as mortality, morbidity, episiotomy rates, cesarean rates, and use of best practices in labor and birth care.

This framework, supported by a practice based on scientific evidence and crossed by individualized, integral, and shared care, ensures the humanization of care. However, it seemed to prevail among managers before participating in the PPA that changes in the physical structure would promote the humanization of care.

*“We had already asked for delivery beds that we had already put in the budget. In short, we were already moinho to be able to attend, in a very timid way, without much ambition […]. I had already visited some other hospitals, and I always brought ideas during my visits. (leader/Coord).*

*“Practically, with the time of one year, before the Adequate Childbirth Program, the hospital started some actions in which we did some small reforms in the pre-birth, to try to leave a little more pleasant and humanized environment.” (leader/Coord).*

Before the PPA, the hospitals had already initiated timid modifications related to the structure, which probably favored the decision-making process of adherence to a more systematized intervention program. However, it is impossible to predict if, already in the decision-making process, there was the maturity of understanding of the institutions concerning the physical structure being only one of the components of the scenario in the humanization of care, and not a defining component.

### The search for improved care

This category was defined by reports relating to efforts that the hospital had begun to execute before the PPA but had not progressed and was aided by the program (Fig. 3). In addition, they reflect on how the form of labor and birth needs to be revised in the private care network.

*“[…] basically our role here, as the leader or head of the service, is that we have a practice in the first place that is safe and a practice that is within the recommendations of the big bodies that govern good practice, among them the World Health Organization.” (Manager).*

*“Not scheduling cesarean sections under thirty-nine weeks, we had already started in the year 2014, because we had already seen by the indicators that we had a high rate of respiratory distress (in newborns), by obstetricians performing cesarean sections in those children that are considered late preterm.” (Manager).*

*“Basically we tried to do prenatal care within the best standards of protocols, bring some degree of preparation to these patients, through courses for pregnant women […]” (leader/Coord)*

*“[…] we needed to change obstetrics in Brazil, especially in private health, because it had become a cesarean factory and that we had abandoned traditional obstetric practices, and normal became cesarean.” (Manager)*

*“[…] our kind of service had been facing difficulties concerning this epidemic of cesarean sections […]we were not knowing, by ourselves, how to dribble these difficulties.” (leader/Coord)*

## Third stage: decision

### The decision to join PPA driven by change agents

The perceived characteristics of the innovation are put to the test when hospital representatives attend the program presentation meeting and return with their relative advantages and possible disadvantages of adopting PPA. This internal “evaluation” process of the hospitals considered compatibility, desire for change, complexity in implementing such an intervention, the perception of women, and health plans in enjoying an interventional innovation, among others.

*“So that is how it happened, so I brought (agents who influenced the decision to join the PPA), I made a report […], and he (the decision agent) understood that we should participate in the program and then, we started.” (Manager).*

*“[…] I had a series of meetings, I showed the Program, I talked to one, I talked to another, until we received the endorsement, the release, from the [decision-making agent], to participate in this Program.” (leader/Coord).*

For this study, we consider as opinion leaders, here represented by influencing agents, those who presented a favorable/disfavorable attitude to the hospital administration regarding adherence to the PPA; as well as change agents, symbolized as decision making agents, actors responsible for the final decision and for signing the term of adherence to the program. Nothing prevents the same person from assuming the two positions; what depends on the degree of management involvement regarding innovation.

## Discussion

Over the years, several normative [22, 23] and formative [24] interventions in the Brazilian public obstetric service have been implemented to reduce cesarean sections and improve women’s experience of delivery and babies’ experience of birth. However, effective interventions to change the care model and consequent change in labor and birth are still considered atypical in the private sector. In 2010, the Public Prosecutor’s Office filed a Public Civil Action [25] against the ANS, an agency linked to the Ministry of Health and responsible for creating standards, controlling and monitoring the health insurance market in the country [2], so that there would be effective measures to reduce unjustified cesarean sections.

Thus, when the PPA comes up with a proposal to change the obstetric care model in private hospitals, it suggests an intersection between health and innovation. Such an intersection is challenged not only by the need to change poor indicators but also by the entrepreneurial vision of the very leaders involved in the adoption of PPA, who must adjust to the needs and expectations of patients, organization professionals, and, most importantly, the institutional mission’s yearning [26].

In knowing about the adherence to innovation, it should be apparent to those involved that the proposed innovation is not a neutral act of people or institutions because it needs to be based on its possible effects to implement it. Thus, identifying the need for the innovation; in which body such innovation will be implemented; whether it will be socially constructed; whether it maintains minimum conditions of access [14]; in addition to not being divorced from tradition or so-called “old innovations” [27], are valid questions in the adherence of health innovations. Such points strengthen aspects that go beyond the interests of capital, taking into account the social interests involved in the contexto [14]. In this way, it supports the findings of this study, which show that the knowledge stage is critical for the appropriation of the PPA and the innovation proposal that the intervention communicates.

One should be careful about the possible adverse impacts when applying innovation in health institutions since it does not necessarily have only positive consequences [26]. Recent studies show that the prioritization of aspects related to assistance and care in private services is minimized to the detriment of the hoteling aspects of maternity hospitals [7, 28]. In addition, the deconstruction and reconstruction of a new paradigm by management and professionals related to control of bodies at the time of labor and birth are challenging and sometimes unacceptable [29]. Modifying the position of labor, centering care on the woman and her family as an institutional philosophy, respecting the moment of birth as individual and unique are changes that challenge the biomedical model [30].

Only the strengthening and empowerment of women is ineffective when the cesarean section has become normalized in Brazil by the thinking and attitude of specialists, which is perpetuated in health institutions as something simple, safe, aesthetic, clean, and painless [28, 29]. Women are subject to impediments that do not facilitate or promote their autonomy [31].

There is, then, a real need for the strengthening and recognition of the intentions that surround private obstetric health institutions in Brazil, conformed by a mission, vision, and philosophy that must dialogue with the wishes of women, with the best scientific evidence, and with the humanization of labor and birth. By joining the PPA, the proposal to change the care model seemed to go beyond building a new professional way of acting, punctuated only by specific training, but also to a follow-up of the changes over time, to the recognition of people strategies that could encourage changes in conduct, practice, and care, envisioning a new institutional and not only professional thinking and positioning [32].

In this sense, finding strategic leaders [33, 34] for the appropriation of the intervention seems to have been a concern of the hospitals. The exciting findings were to identify the medical professional as a reference for the perpetuation of the intervention during the implementation in the Brazilian private hospital scenario. It is valid to point out that the social respect in the country correlated with the medical professional is rooted in the biomedical model, currently much questioned [35].

However, it was noticed that the choice of this change agent in charge of sustaining the intervention was influenced not only by his professional knowledge and technical abilities but also by his institutional influence over the remainder of his category and other professional categories [36, 37]. Therefore, the choice of the change agent to know and persuade about the proposed innovation may be one of the crucial factors not only for adherence and implementation and also for the sustainability and reinvention power of the innovation.

## Conclusion

The adherence to the Adequate Childbirth Program went through the appropriation of the innovative intervention to then adopt it. The professionals representing the institutions, during this decision-making process, felt privileged to have received exclusive invitations to participate, sent by the organizers of the program. This simple act expresses special care, identifying each service as unique.

The lack of multifaceted obstetric interventions in the Brazilian private service, the social and market status, the search for better care, and the possibility for hospitals to carry out structural reforms by being inserted in the intervention motivated adherence to the PPA. The use of agents of influence to convince peers was key to the final decision to join.

The speech place of the persons interviewed is an essential factor to consider since it might limit the facts in their natural context to match the research institution’s expectations. Furthermore, the data was obtained after the participants had joined the program, favoring possible memory biases during data collection. Finally, there was a gap between the time the data was collected and its analysis. Our research, however, revealed the logic of a work shaped by institutional, social, and cultural rules that do not change quickly, that require time, desire for change, and training to improve care, which reinforces the validity of this study.

Journals rarely publish the point of view of management and coordination professionals in the innovation decision-making process. The focus often permeates the deployment results. In this research, we have the opportunity to deal with what motivated midwifery managers and leaders/coordinators in the Brazilian private sector to seek a change in the care model.

This fact reveals that to continue reducing cesarean sections in complementary institutions in Brazil, complex interventions, such as the PPA, are needed to ensure better results. Another fact revealed by this study permeates the transmission of knowledge about PPA, having occurred strategically by members of institutions considered socially representative and respected in the professional field, in this case, the physician. This may allow a greater adherence of professionals. However, we still do not know if this fact revealed greater assurance in the implementation and sustainability of the program in these institutions.

## Data Availability

The datasets used during the current study are available from the corresponding author on reasonable request.

## Abbreviations

ANS: National Supplementary Health Agency
CEO: Chief Executive Office
CS: Cesarean Section
ENSP: National School of Public Health
FIOCRUZ: Oswaldo Cruz Foundation
IHI: Institute for Healthcare Improvement
MH: Ministry of Health
PPA: Adequate Childbirth Program (“Projeto Parto Adequado”)
SUS: Unified Health System
WHO: World Health Organization
